# The Agile Deployment of Machine Learning Models in Healthcare

**DOI:** 10.3389/fdata.2018.00007

**Published:** 2019-01-08

**Authors:** Stuart Jackson, Maha Yaqub, Cheng-Xi Li

**Affiliations:** Analytics Center of Excellence, IBM Watson Health, Ann Arbor, MI, United States

**Keywords:** agile, analytics engineering, continuous delivery, health informatics, machine learning

## Abstract

The continuous delivery of applied machine learning models in healthcare is often hampered by the existence of isolated product deployments with poorly developed architectures and limited or non-existent maintenance plans. For example, actuarial models in healthcare are often trained in total separation from the client-facing software that implements the models in real-world settings. In practice, such systems prove difficult to maintain, to calibrate on new populations, and to re-engineer to include newer design features and capabilities. Here, we briefly describe our product team's ongoing efforts at translating an existing research pipeline into an integrated, production-ready system for healthcare cost estimation, using an agile methodology. In doing so, we illustrate several nearly universal implementation challenges for machine learning models in healthcare, and provide concrete recommendations on how to proactively address these issues.

## 1. Introduction

Contemporary software engineering is driven by a number of key themes, such as agile development cycles and the continuous delivery of production software (Fowler and Highsmith, 2001; Shore and Warden, 2008). Such approaches allow for neater partition of development work related to current and future software capabilities, and help to streamline maintenance flows, product documentation, and development team communication. Unfortunately, the continuous deployment of predictive analytics is often hampered by poorly thought-out maintenance plans and non-agile methods of deployment, a phenomenon experienced across widespread industries (Demirkan and Dal, 2014), including in health informatics settings (Reeser-Stout, 2018). In this short Perspective, we describe our team's ongoing efforts and the lessons learned so far in the agile deployment of a new predictive analytics model related to healthcare cost estimation. While this model is designed for a specific use case (i.e., predicting cost in the US Medicaid population), our integrated deployment strategy is more general, and could transfer easily to other claims-based models.

We begin below with a brief overview of the typical challenges and maintenance issues experienced when deploying machine learning models in health informatics settings, using actuarial models as an example. We then describe our use of agile methods in a new actuarial product deployment, emphasizing the hybrid nature of agile data science, the important concepts of iteration and experimentation, and the unique challenges faced and solutions developed to fulfill key product requirements. Along the way, we provide a high-level description of the model that was trained and productionized, and an illustration of how internal maintenance and client use can occur side-by-side in the integrated production codebase. Finally, we conclude by providing some general recommendations for hybrid development teams to consider when tasked with developing and deploying a new healthcare analytics product.

In passing, note that an earlier version of the research model we deployed was developed by a separate team at IBM, and has been described in detail in their separate methods paper (Ramamurthy et al., 2017). As such, we refrain from discussing this model from a deeper research or design perspective. Our aim in this short Perspective is to describe the challenges faced in refining and productionizing one instantiation of a research model, and the considerations required in sustaining continuous delivery and internal maintenance of a new healthcare analytic. In the spirit of continuous delivery, these efforts are necessarily ongoing.

## 2. Actuarial Models in Healthcare

Accurate healthcare cost estimation is of critical importance to medical organizations, governments, and societies at large, with healthcare expenditures a primary drain on public resources worldwide. The availability of reliable cost estimates for a population can aid insurance plan administrators and other healthcare professionals in effective resource planning, in risk adjustment, and in developing strategies for population health management (Duncan, 2011). A wide variety of predictive algorithms have been developed over the years for estimating healthcare costs from administrative claims data, including numerous proprietary models (Winkelman and Mehmud, 2007). These models are often tailored for very specific patient populations, use cases, or input data needs; yet, a common goal of such models is the prospective identification of future high-cost claimants, often using linear or tree-based regression methods (Meenan et al., 2003; Bertsimas et al., 2008).

While the development of a high-quality risk model is itself a challenge, the successful, long-term deployment of such a model in applied settings is equally challenging, requiring careful consideration of the potential maintenance issues that could arise from changing industry, client, or technical needs. For example:

- Regular (e.g., yearly) updates might be required when new training or scoring data become available
- Irregular updates might be required when industry reference files (e.g., ICD diagnosis codes) change
- Minor model improvements or technical corrections and bug fixes might be required on an *ad-hoc* basis
- Major model improvements or new functionalities might be required subject to evolving client needs
- Changes to deployment hardware or other architectural constraints may need to be accommodated

Following the completion of any such maintenance task, an additional period of code review, model retraining, and product testing might also be necessary. Yet, the typical research model lacks the continuously-integrated organization necessary to efficiently handle such common maintenance issues. Below, we describe how our team adopted an agile framework in deploying a new actuarial model into production, streamlining the model training, and production process to support effective continuous delivery.

## 3. Principles of Agile Data Science

### 3.1. Avoiding the “Pull of the Waterfall”

Our product team was tasked with training and deploying a new claims-based risk model, which we approached initially from an agile framework. Agile software development practices are now industry standard, supporting efficient methods of collaboration and effective ways of getting work done (Fowler and Highsmith, 2001; Shore and Warden, 2008). As the field of data science evolves, however, it is increasingly clear that existing agile methods will need to adapt to successfully support this hybrid development domain (Jurney, 2017; Reeser-Stout, 2018). For example, the typical time allowed for a research data science project conflicts sharply with the standard agile development cycle. This can have the effect of forcing otherwise agile predictive analytics projects toward more sequential development cycles–the so-called “pull of the waterfall” (Jurney, 2017). This can be particularly problematic in healthcare research and product work, where additional constraints are often at play (e.g., strict data access rules).

**Figure 1:**
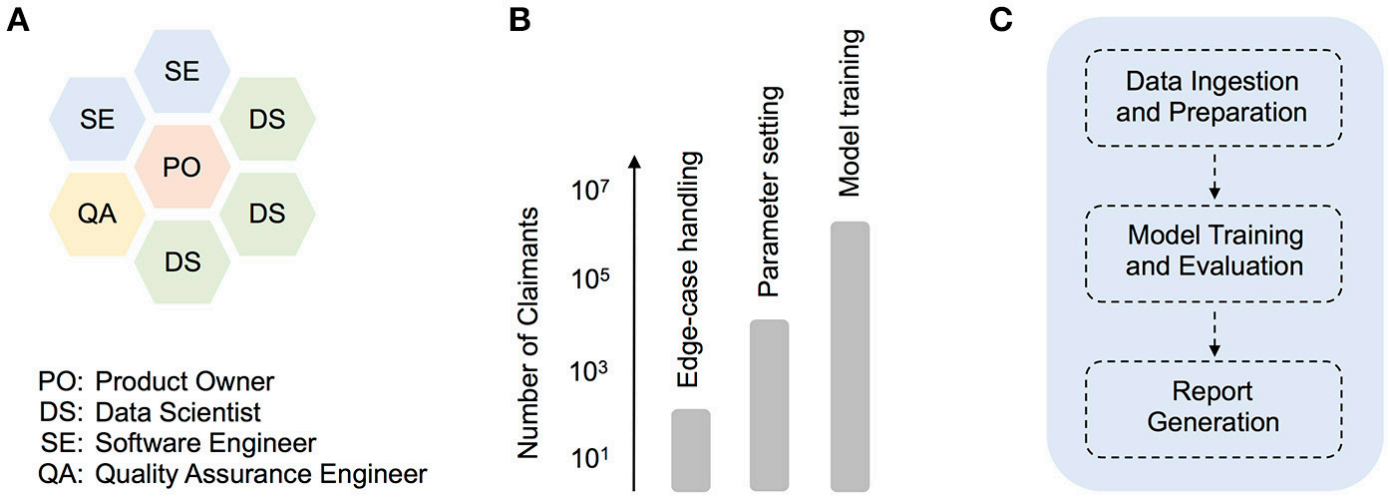
The agile deployment of machine learning models in healthcare. **(A)** Organization of roles in our hybrid data science development squad, **(B)** Synthetic illustration of how different sets of production tasks typically warranted working with claimant dataset sizes of a particular scale, from technical debugging with smaller input datasets, to product model training with large-scale input data, **(C)** Schematic depicting higher-level modularization within the core model pipeline.

To avoid the sequential handover of work from one group (e.g., analytics) to another (e.g., engineering), we established early on a hybrid development squad (Figure 1A), which facilitated the direct interaction between data scientists, software engineers, a quality assurance (QA) engineer, and a product owner. We also adopted the development and deployment terminology common in software engineering. For example, as a given development phase was completed, our product code was scheduled to pass first to a QA testing phase (or “TST”), then onto user-acceptance testing (“UAT”), and only then into production. While such terminology is somewhat foreign to many research data scientists, these methods prove essential in a highly collaborative, production context. In contrast, prior attempts at formalizing methods for predictive analytics development, such as CRISP-DM (Shearer, 2000), are too far removed from contemporary software engineering practices, having little to say about code deployment or collaboration across multi-functional (and often remotely-located) teams. That being said, there is enormous potential for the refinement of improved hybrid agile methodologies that more smoothly integrate with standard research science practices. Below, we describe one such hybrid strategy that we adopted during our product deployment.

### 3.2. Iteration and Experimentation

Our product team actively experienced the conflict between research and software development worlds, learning the hard way that specifying a final production date ahead of time is often incompatible with doing successful, agile data science (Jurney, 2017). To overcome this conflict, we evolved a process that roughly centered around two key ideas–*iteration* and *experimentation*. In the spirit of agile, our iterative process encompassed groups of tasks completed over individual three-week sprints, often involving extremely dynamic code changes that ranged across the entire product pipeline. As a rule-of-thumb, we aimed to not only have the code running end-to-end at key iteration milestones, but more importantly, to better understand the data flow and model behavior at key points in the pipeline. Only then was the model pipeline deemed worthy of intermediate delivery to other users (e.g., QA engineer).

As iterations progressed, however, the importance of parallel experimentation quickly became apparent, both from a technical debugging and model training perspective (Figure 1B). For example, when building a client-facing product that deals with medical claims data, comprehensive edge-case handling is a particularly challenging technical issue. In our case, smaller input datasets often included blank or missing claims data for time ranges that the model expected (i.e., empty months), and revealed bugs in parts of the original pipeline dealing with the aggregation of disease and cost information. Given the numerous time-related, cross-dependencies in the prediction pipeline, the development of appropriate code fixes benefited greatly from over-and-back interactions with a QA engineer, and trial code runs with smaller datasets of varying size (e.g., 10^1^ or 10^3^ claimant records). In general, experiments with smaller dataset sizes were essential throughout development, for performing quick, technical debugging. As the product code was better refined, experiments on larger datasets (e.g., 10^5^ claimant records) allowed for deeper code and model understanding (e.g., parameter exploration and tuning). Finally, as a given release date approached, production model fits were carried out on a formally-curated, large training dataset, with up to several million unique claimant records (Figure 1B).

### 3.3. Reinforcing the “Hybrid” Nature of the Work

How successful was this hybrid agile approach from a data science perspective? All of the data scientists agreed that this more foundational, agile approach to development provided clear advantages over isolated development styles, removing crucially the need for analytics developers to deliver code to a separate production team in a sequential fashion. The hybrid approach was self-reinforcing, in the sense that it encouraged all squad members to play multiple, interacting roles throughout development. For example, while data scientists played the major role in finalizing the core prediction model (described in detail later), the QA and software engineers had numerous opportunities to examine and refine this code, providing complementary feedback which improved the overall quality of the data science work. Likewise, while the QA and software engineers were primarily responsible for smooth deployment of the end-to-end pipeline (described in detail later), the data scientists spent substantial time facilitating this process through proper packaging and documentation of code. This facilitated the deployment process, and again, involved constant cross-squad interaction and feedback. The end results were successful, iterative deployments of the full codebase into production.

One broader advantage of this hybrid system is the ability to more easily organize data science projects at the level of multiple squads, thereby maximizing resource use and collaboration potential. For example, the hybrid squad described here (aka *Mercury*), worked independently of several other “planet” squads (e.g., *Jupiter*), although with some higher-level direction from a scrum master working across multiple teams. While this type of organization is already common in traditional software engineering environments (e.g., tribes, chapters, etc.), we believe it requires the creation of truly hybrid squads, involving both data scientists and software engineers, to be successful in an analytic development context.

## 4. Challenges in Analytic Development

### 4.1. Providing Multiple Model Types in One Platform

We now describe in detail several key requirements of the software development product, and the associated development and coding challenges we faced. In a following subsection, we illustrate how we tackled these problems and gauged the success of the improved processes.

The key requirements of our analytic product related to the functionality to provide access to multiple model results for a single input claims dataset. Specifically, actuarial predictions for up to twelve model variants were necessary (Table 1), with models varying in terms of the time period of prediction (e.g., concurrent year vs. prospective year), the cost components being predicted (e.g., medical costs only vs. medical and pharmacy costs), and the form of thresholding or truncation applied to outliers (e.g., no truncation vs. $100k or $250k truncation). These model variants were selected to support diverse risk-adjustment use cases, from the retrospective measurement of provider performance (e.g., using concurrent year models), to the estimation of a population's future healthcare costs for resource planning purposes (e.g., using prospective year models). While the original research model we inherited provided some powerful functionality in this regard, there were numerous engineering challenges to face from a production and deployment perspective. Crucially, several of the capabilities below had to be productionized to work identically in both training and deployment (i.e., scoring) scenarios, thereby supporting a more easily maintainable codebase and product. For example:

- The product training data cohort needed to be defined, and needed to accommodate all twelve model conditions, in terms of the time range of claims data, pharmacy costs availability, and the exclusion of certain data sources with unreliable or incomplete costs (e.g., capitated health plan data, claimants that are dual-eligible for Medicare, etc.). An inadequetely-defined training cohort would negatively affect the functioning of all of the data ingestion, preparation, and scoring modules (described in detail later), and the lack of a cohort definition module would make all future updates to the product unnecessarily cumbersome.
- The functionality for selecting and running only a subset of models (based on user input) needed to be developed. While input requests to run the analytic were initially sent in one-at-a-time, along the way it was decided that the end user should have the ability to run multiple versions of the model in one request (e.g., all twelve variants or only a subset). For example, dependent on the particular risk-adjustment use case, an end user might request results from only the concurrent year models, or only those with a specific truncation threshold (e.g., $100k).
- The flexibility to change key model parameters automatically and “online” (i.e., during actual training or scoring) was similarly required. In some instances, these changes involved relatively minor parameter updates (e.g., switching from one truncation threshold to another). For others, substantial pipeline rerouting was necessary (e.g., switching from local directory operations during training to directory operations which are dynamically set during deployment).

**Table 1:** Parameters of the twelve model variants deployed to fulfill diverse end-user requirements.

**No**.	**Model type**	**No**.	**Model type**
1	Concurrent, Total Cost	7	Prospective, Total Cost
2	Concurrent, Total Cost ($100k)	8	Prospective, Total Cost ($100k)
3	Concurrent, Total Cost ($250k)	9	Prospective, Total Cost ($250k)
4	Concurrent, Medical Cost Only	10	Prospective, Medical Cost Only
5	Concurrent, Medical Cost Only ($100k)	11	Prospective, Medical Cost Only ($100k)
6	Concurrent, Medical Cost Only ($250k)	12	Prospective, Medical Cost Only ($250k)

*“Concurrent” models predict annual healthcare costs for the same 12-month period as the input data; “Prospective” models provide predictions for the subsequent 12-month period (i.e., next year). Note that ‘Total Cost' refers to models that predict combined medical and pharmacy costs, and that the dollar values in brackets refer to truncation thresholds applied to outlier claimant data. See text for further details*.

### 4.2. Solutions That Satisfy the Key Product Requirements

How did we tackle these challenges, and how did we measure the success of the resulting processes? By implementing a variety of sustainable coding practices, we developed solutions to these issues as follows:

- To ensure integrity of our training cohort, we first developed a formal “cohort definition plan” (similar in spirit to a CONSORT diagram). This plan involved several stages, including the key steps of: (a) selecting a large random sample of patient IDs (e.g., 5 million) covering a time range of interest (e.g., 2013-2017); (b) excluding those subset of IDs linked to capitated health plans or having dual-eligible for Medicare status (as claims costs from these subgroups of patients are often incomplete); (c) extracting the complete enrollment and medical claims data for all remaining valid patient IDs.
- This plan was then implemented in a series of code modules, used to extract data from internal, proprietary Medicaid databases. To verify the success of this implementation, we monitored the smooth running and completion of the data extraction code, and ensured that the resulting cohort data mapped correctly to a formal data dictionary that we had prepared. The data dictionary in particular acts as a fundamental reference file for all users of the production analytic, and was a crucial milestone in our development. With minimal modifications, the overall cohort definition module can be used in future analytic developments (e.g., after new training data is obtained or industry reference files are updated).
- To ensure that the analytic had the functionality to take specific user requests and to update parameters “on the fly,” we began by making the decision to keep the code as flexible as possible and not to hardcode parameters for any of the model variants. Our team then developed a series of input-level scripts (primarily in shell scripting languages), as well as later module-specific templates (in Python), that updated dependent on the specific user input. For example, if the user requested results for models with $100k truncation only (models 2, 5, 8, and 11; see Table 1), the analytic proceeded to automatically update relevant parts of the code and configuration files for each of these models in turn.
- After finalizing these cohort definition and model specification techniques independently, the overall set of solutions was tested through extensive model running at key iteration milestones (e.g., during quality assurance and user-acceptance testing). The above functionalities, which served to provide multiple model types in one platform, were successfully deployed in each of our iterative releases.

## 5. Deploying an End-to-End Solution

### 5.1. The Core Model Pipeline

We deployed an end-to-end healthcare cost estimation solution that can be maintained internally with relative ease (i.e., recalibrated or extended in functionality), and continuously pushed to cloud production environments where client scoring can occur. The product we deployed was refined from a previously developed research pipeline, described in detail elsewhere (Ramamurthy et al., 2017), and contains a family of cost models that we trained on Medicaid claims data. As described earlier, models varied along a number of parameter dimensions, including the time period of prediction (e.g., concurrent year vs. prospective year), the cost components being predicted (e.g., medical costs only vs. medical and pharmacy costs), and the form of thresholding or truncation applied to outliers (e.g., no truncation vs. $100k or $250k truncation). Model inputs included basic demographics (e.g., age and gender), enrollment details (e.g., number of months enrolled), and diagnosis information. In passing, note that while we avoid discussing Medicaid data in detail here, focusing instead on our general agile development framework, the interested reader can find numerous sources discussing specific disease prevalence and hospitalization issues in these claimant populations e.g., Trudnak et al. (2014).

To facilitate maintenance and future development, the pipeline utilizes a core set of code modules, which at a high-level, perform essentially three functions (Figure 1C). First, a sequence of *data ingestion and preparation* modules read in the required input files (including enrollment, claims, and auxiliary input files), and perform batch processing on these in order to aggregate raw input data into intermediate database tables. Operations such as dummy-encoding, feature enrichment, and sparse matrix creation are also carried out at this stage, to improve the efficiency of later data handling. In the second high-level phase of processing, data is passed to modules that support *model training and evaluation*. The data subsets required for training or evaluation are isolated at this stage, and in the case of internal training, a multi-stage regression model is trained and saved. Model parameters can be configured in advance using configuration files. Model evaluation or scoring is then performed, either on a separate test dataset (in internal training mode) or directly on client data (in client scoring mode). Finally, in the *report generation* step, patient-level predictions (e.g., costs and risk scores), as well as overall model performance reports, are saved to file.

### 5.2. The Integrated Product Codebase

Refining a product codebase is a collaborative effort involving multiple people contributing to the same overall product vision. To make this happen, it is important to host the code in proper software deployment platforms, and to use technologies that support efficient collaboration. This helps to ensure that maintenance tasks can be carried out easily without affecting the core pipeline. For example, changes to industry reference files (e.g., ICD diagnosis codes) or other evolving industry requirements (e.g., the use of social determinants information) can be smoothly incorporated into the product data model and modular pipeline. The integrated product codebase and deployment process that we refined allows for easy modification of code components and model recalibration, without significant effects on code integration and product delivery. Below we describe key characteristics of this integrated product pipeline.

The integrated product codebase is defined by three key platform characteristics–*version control, containerization*, and *continuous integration*. First, by refining the final codebase in an environment that supports version control (e.g., GitLab; https://about.gitlab.com), we ensured that every team member had access to and could modify the same codebase. This facilitated efficient collaboration on the final product, and limited the need for having standalone versions of the code existing in different places. Second, to control the vast array of packages required in code running, we adopted a containerized approach to code delivery. Even a single faulty or missing package can cause critical breakages in a code pipeline similar to the one we deployed. To avoid this problem, container technologies (e.g., Docker; https://www.docker.com) allow one to host code in a virtual environment that has all the required software packages pre-installed. This approach facilitated deployment on a production server and eliminated the need for team members to individually sift through package installation requirements, saving a considerable amount of time. Finally, the pipeline was integrated by software engineers into final testing and production layers, with the aim of automating the code building and establishing continuous integration of the product. Open-source continuous integration tools (e.g., Jenkins; https://jenkins.io) allowed the team to monitor the code deployment in real-time and quickly identify any defects.

## 6. Conclusion and Key Recommendations

We provided here a brief overview of our attempts at refining an agile data science methodology to support a new healthcare analytic deployment, emphasizing the hybrid nature of agile data science and the important roles played by team iteration and model experimentation. There is clearly enormous potential for the development of more formal approaches to agile data science, both in healthcare and elsewhere, which we hope this brief overview has illustrated. As a starting point, we provide the following general recommendations, when faced with the challenges of any new healthcare analytic deployment:

- Track your work: Incorporate a formal agile tracking tool into your work from the outset, and organize each piece of your work into a separate “user story.” Tracking systems encourage teams to remain actively engaged and to communicate clearly, behaviors which are particularly important in hybrid teams, where skill sets might overlap less than in traditional software engineering teams. In addition, agile approaches encourage the use of the “backlog” to keep track of upcoming tasks, as not every feature or user story will be complete for a given product release. We recommend using it. For example, at a preliminary milestone in our development work, non-critical aspects of the output file formatting were not fully finalized; by adding appropriate notes to the backlog, the team was able to more easily monitor the status of this and other remaining tasks across iterations.
- Investigate and implement: For some data science issues (e.g., finalizing production parameter settings), it is important to allow sufficient time for problem understanding. We have found it beneficial in such cases to pair “investigation” and “implementation” user stories. What do we mean by this, and why is it important in a data science context? The purpose of creating an investigation user story is to allow the team sufficient scope and time to research complex model details, and thereby more clearly define the ideal boundaries of the prediction pipeline. The specifics of a computational model are often more nuanced in implementation than traditional software components, and implementation errors often have subtle effects that are difficult to detect. In one example, our squad investigated methods for handling the well-known medical claims “run-out” issue (i.e., the time lag between recent medical services and subsequent claims payment processing, which can be several months in duration). After detailed investigation, we developed a plan in sync with industry standards, accommodating the processing of relevant medical claims paid within a 3-month run-out window after the end of a claim year. By performing and closing out this investigation story, team flow was at least maintained, even if production code was not necessarily updated significantly. We then implemented this plan in a separate user story, and measured the implementation success on small samples of data, by comparing aggregated claims costs from the pipeline to manually aggregated costs. We believe this “investigate then implement” approach to work definition is particularly useful in a hybrid squad context, as it reinforces continual communication and transfer of learning throughout the diversely-skilled squad.
- Release in increments: Develop an incremental product release strategy, and communicate this plan clearly and early to others. This should help in ensuring that realistic deadlines are formed, and that these are driven primarily by the team's estimation of the workload (not by external stakeholder needs). More specifically, the release process should comprise of a strategic set of deadlines which cater appropriately to development team resources and incremental product release goals. For example, in our development work, we first scheduled an early or “Beta” release directed toward an internal client. By doing this, the deployed codebase was put through the standard release testing processes (e.g., quality assurance and user-acceptance testing), without any changes or biases introduced by the data science team for a defined period of time. This allowed time for independent feedback regarding the codebase and for completion of remaining backlog tasks (e.g., improvements to the formatting of output reports). It also allowed for fine-tuning and retraining of the core prediction model on a larger training dataset, thereby improving the overall performance and quality of a later “Production” release.

In conclusion, we believe the hybrid squad model has many benefits over isolated teams when doing data science software development. In addition to improved communication and collaboration, as well as the removal of sequential handover of work, the hybrid squad model provides substantial opportunity for skills transfer and innovation that would otherwise not occur. While it is still early days for the hybrid squad system we have described, the potential has been obvious to everyone involved, including at the team management level. That being said, the recommendations above illustrate some likely areas of difficulty for new hybrid squads, which we suspect will typically arise in setting sensible release strategies and deadlines. Yet, we firmly believe that hybrid analytics teams are the future, and sincerely hope that others can build on the recommendations outlined here.

## Data Availability Statement

No datasets were generated for the purpose of writing this manuscript, and all relevant information is contained here. Proprietary code referred to in the manuscript is not publicly available (property of IBM).

## Author Contributions

All authors contributed to the conception and design of the paper. SJ wrote the first draft, and MY, C-XL, and SJ wrote additional sections. All authors contributed to manuscript revision and approved the final version.

### Conflict of Interest Statement

At the time of original drafting, the authors were all full-time employees of IBM. The authors share this perspective with the sole aim of contributing to open dialogue on the topics described in the manuscript.
